# Design of a care pathway for Hepatitis C: a pilot study with three University Hospitals from Tuscany

**DOI:** 10.1093/eurpub/ckac131.400

**Published:** 2022-10-25

**Authors:** I Corazza, L Ceccarelli, G Moretti, L Tavoschi, M Vainieri

**Affiliations:** 1 Health and Management Laboratory, Sant'Anna School of Advanced Studies, Pisa, Italy; 2 Faculty of Medicine and Surgery, University of Pisa, Pisa, Italy

## Abstract

Monitoring and evaluation activities are recognised as key to quality improvement in healthcare performance. The present study is intended to design a performance evaluation system for care pathway for patients with chronic Hepatitis C virus (HCV) infection, to follow them along the continuum of care throughout regional healthcare services, from diagnosis to treatment course completion. Four phases of the care pathway, namely diagnosis, linkage to care, treatment, and outcome were identified. Each phase of the care pathway was populated by a set of observation and evaluation indicators. Data sources were: administrative health data from the Tuscany Regional Healthcare System; patient-reported experience and outcome measures collected by means of questionnaires administered by the health professionals during patients’ consultation in the three University Hospitals of the Tuscany Region. The availability of data, collected from the administrative flows and thanks to the active involvement of health professionals, showed the feasibility of designing a care pathway for HCV. More particularly, using administrative data, three performance indicators were calculated for the prevention phase, two for the linkage to care phase and two more for the treatment and outcome phases, respectively. Moreover, two indicators related to linkage to care and outcome phases were designed, but data require further investigation. On the other hand, using patient-reported experience data, four indicators can be calculated for the linkage to care phase, while regarding patient-reported outcomes, the feasibility of calculation depends on the number of patients that will be involved in follow-up visits. The care pathway designed may be useful to: identify shortcomings of the healthcare services for chronic HCV patients; foster quality improvement actions; inform allocation of resources to accelerate HCV elimination in Tuscany.

**Key messages:**

• The authors propose a care pathway for Hepatitis C, consisting of four distinct phases, populated respectively with diagnosis, linkage to care, treatment, and outcome indicators.

• The care pathway can be used as a management tool for the identification of possible quality improvement actions to be undertaken with respect to the healthcare services provided to HCV patients.

